# Choosing treatment with an elderly patient having a distal radius fracture

**DOI:** 10.2340/17453674.2026.45945

**Published:** 2026-06-22

**Authors:** Marcus LANDGREN, Cecilia Mellstrand NAVARRO, Teemu KARJALAINEN, Rikke THORNINGER, Sondre HASSELLUND, Rasmus Wejnold TROEST, Jeppe Vejlgaard RASMUSSEN, Hebe Désirée KVERNMO, Dennis Winge HALLAGER, Tamara ROZENTAL, Magnus TÄGIL

**Affiliations:** 1 Department of Orthopedic Surgery, Copenhagen University Hospital, Herlev and Gentofte, Gentofte; 2 Department of Clinical Medicine, University of Copenhagen, Copenhagen, Denmark; 3 Department of Clinical Science and Education, Södersjukhuset, and the Department of Clinical Sciences Danderyd Hospital, Karolinska Institutet, Stockholm, Sweden; 4 Hospital Nova of Central Finland, Jyväskylä, Finland; 5 Department of Orthopaedics, Aarhus University Hospital, Aarhus, Denmark; 6 Division of Orthopaedic Surgery, Oslo University Hospital, Oslo; 7 Institute of Clinical Medicine, University of Oslo, Oslo; 8 Department of Orthopedic Surgery, Hand Surgery Unit, University Hospital of North Norway, Tromsø; 9 Institute of Clinical Medicine, University of Tromsø – Arctic University of North Norway, Tromsø, Norway; 10 Department of Orthopedic Surgery, Zealand University Hospital, Køge, Denmark; 11 Harvard Medical School. Beth Israel Deaconess Medical Center, Hand and Upper Extremity Division, Boston, MA, USA; 12 Department of Clinical Sciences, Lund University, Lund; 13 Department of Orthopaedics, Skåne University Hospital, Lund, Sweden

## Abstract

Distal radius fractures in adults primarily occur in the elderly, in whom comorbidity, polypharmacy, dependency, and limited functional demands often coexist. Most patients have been managed non-surgically with casting. However, only a few trials with high evidence exist on comparing non-surgical and surgical treatments in the elderly. Given the heterogeneity of the aging population, a universal approach to treatment selection may not be feasible. This educational article aims to discuss the advantages and disadvantages of non-surgical vs surgical management, radiological assessment, complication risks, and osteoporosis screening. Moreover, we suggest surgical technical tips, a treatment algorithm, and a decision-making strategy that considers both functional demands and individual needs.

Distal radius fractures (DRFs) are among the most common injuries in orthopedics, yet a gap remains between evidence and clinical practice. Half of these fractures occur in persons > 65 years and 20% in those > 80 years [1,2]. There is no universal definition for when a patient becomes old, therefore the definition of an “elderly patient” is challenging. The proportion of patients undergoing surgery has increased, reaching a plateau in recent years of approximately 20–25% in Finland, Denmark, and Sweden [1-3]. The type of surgical management has also shifted; volar locking plates (VLPs) have replaced percutaneous pinning and external fixation [2,4]. In current DRF guidelines, age 65 years and older is often used as a proxy for low functional demands, and chronological age has strongly influenced the choice between non-surgical and surgical management [3,4]. However, in an otherwise healthy and active elderly patient, a DRF may represent the only limiting condition, hence treatment strategies used in younger adults may be appropriate for some elderly individuals [5,6]. In contrast, in frail patients, burdened by multiple comorbidities and polypharmacy, surgical treatment is less beneficial [7]. Furthermore, the clinical diversity across studies, including population, fracture pattern, and treatment, may compromise their external validity.

The aim of this educational article was to support the treating physician in communicating with elderly individuals regarding treatment choices in shared decision-making. We focus on indications for non-surgical and surgical treatment, potential complications, health-related outcomes, and osteoporosis screening. Considering all these aspects, together with the patient’s functional demands and the individual patient’s needs, we propose a treatment algorithm and a decision-making strategy for the elderly with DRFs.

## Methods

We conducted an updated literature search of Medline, Embase, and CENTRAL based on a previous systematic review and meta-analysis [6] to capture RCTs published after the search date September 2, 2022 comparing surgical treatment using VLPs with non-surgical management of distal radius fractures. Beyond the 12 RCTs included in the systematic review [6], 5 additional RCTs were found and included [8-12]. 3 long-term follow-up studies of previously published RCTs were not included [13-15]. We identified national clinical guidelines for the management of DRFs [16-22]. Further, observational studies reporting on risk of complications, adverse events, osteoporosis screening, and health-economic perspectives were identified through an additional search of Medline, Embase, and CENTRAL, as well as from reference lists. The final search was conducted on October 1, 2025.

### Assessing stability of DRFs

**Figure 1:**
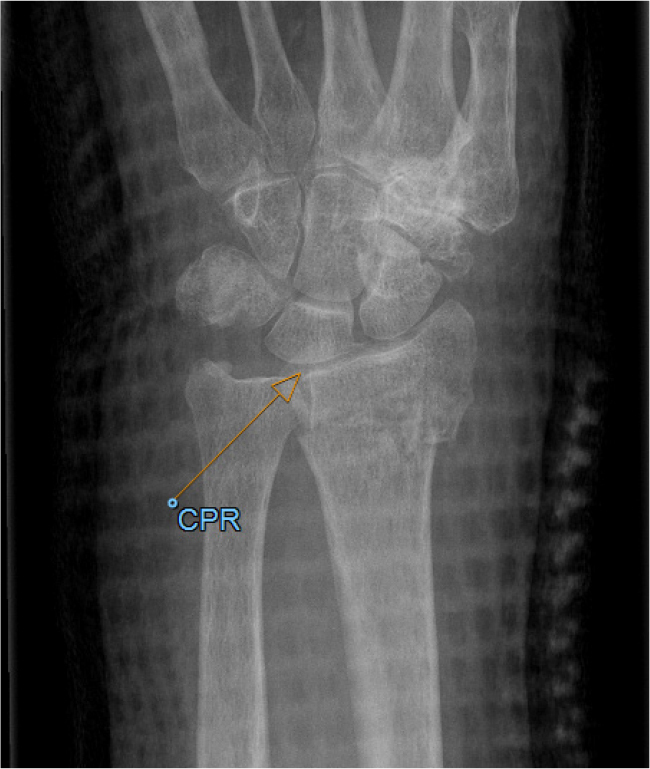
The central point of reference (CPR) is the midpoint between the volar and dorsal articular corners of the sigmoid notch on the radius and can be used in measurement of the radial inclination and ulnar variance.

**Figure 2:**
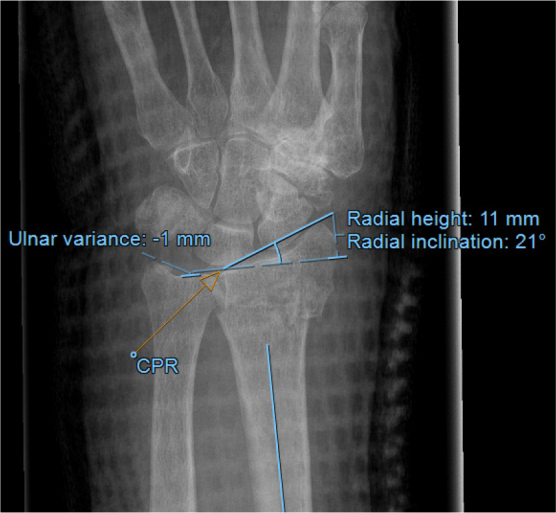
Radial inclination is measured as the angle between the radial styloid and the CPR. Ulnar variance (positive or negative) is the length between the CPR and the distal articular surface of the ulna. Radial height is the distance between the tip of the radial styloid and a perpendicular line to the long axis of the radius.

**Figure 3:**
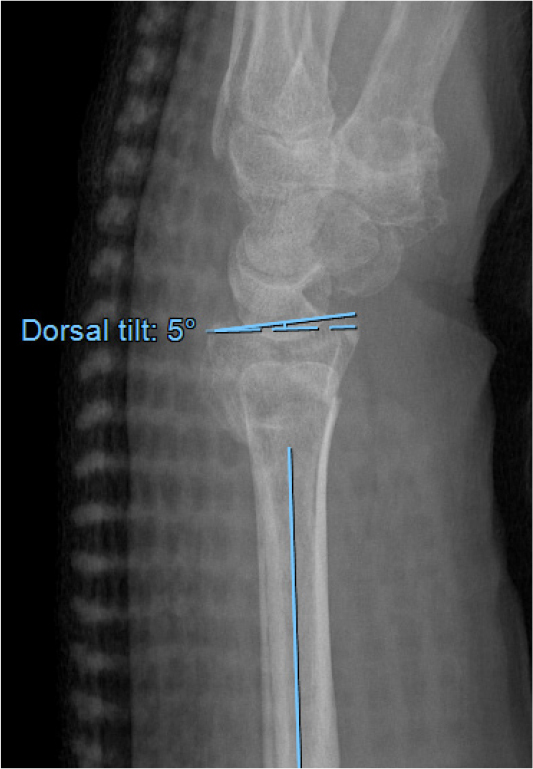
Dorsal tilt is measured as an angle between the volar and dorsal rims of the radius to a line perpendicular to the longitudinal axis of the radius.

The fracture displacement, comminution, intra-articular involvement, and associated ulnar fracture can be the instability predictors. Minimally displaced DRFs are defined as < 10° of dorsal tilt, < 2 mm of positive ulnar variance, or > 15° of radial inclination, as in Figures 1 to 3, illustrating frequently used radiographic measurements [23]. These fractures are typically managed non-surgically, with or without reduction, and cast immobilization followed by 7-to-10-day radiographic follow-up [18,19,22]. Approximately 50% of the DRFs are unstable and will re-displace after cast treatment despite correct post-reduction alignment [24]. Fracture stability can be predicted using the Edinburgh Wrist Calculator [25], based on equations from a cohort of 4,000 fractures [26]. Age emerged as the strongest predictor of instability [26], though the model explained only about 10% of the total variance in displacement risk. The tool can be applied to initially displaced fractures and appears to outperform clinicians in predicting fracture instability [27]. Recent additions to tools for instability prediction include the volar hook; alignment of volar cortices [28]; volar comminution [29]; and marginal displacement between post-reduction and follow-up radiographs [30].

### Correlation between radiographic alignment and clinical outcome

The fracture morphology of DRFs is often considered a predictor of the functional outcome. Among the radiological measurements, dorsal tilt and ulnar variance/radial shortening have shown the strongest association with PROMs in adults. A meta-analysis demonstrated better functional outcomes at 1 year in patients with “acceptable” vs “unacceptable” alignment. However, the differences were not larger than the minimally clinically important difference (MCID) [31], and unacceptable alignment appears to be well tolerated in many elderly individuals. Among the included studies, thresholds for acceptable alignment ranged from 10–20° of dorsal tilt and 2–5 mm positive ulnar variance/radial shortening. While patient-reported function generally improved between 1 and 3 years, a small but statistically significant association was observed between greater dorsal tilt and poorer functional outcomes in the elderly patients aged 70 years and older [13]. A recent study identified a non-linear relationship between radiographic alignment 12 months after the fracture and functional outcomes in adults up to 75 years of age. Poorer outcomes were associated with increasing dorsal tilt, which accounted for 5% of the variability in the measured functional outcome. The decline in clinical outcomes began already at 5° of dorsal tilt but reached the previously established MCID of the 10 scale point at 20° of dorsal tilt [32]. Increasing age is associated with greater tolerance of malunion [33], but a precise clinically meaningful threshold has not yet been established.

**Take-home messages: General considerations and tips**

**• Classification**

Fractures can be judged radiographically as stable, potentially unstable, and unstable according to displacement, comminution, fracture pattern, and degree of osteoporosis. Furthermore, fractures can be classified using the AO classification [56] from a research perspective, and the 3-column theory for understanding and planning the fixation of comminuted fractures [57].

**• Treatment of non-displaced DRFs**

Non-displaced DRFs are immobilized in plaster, which provides effective pain control. Positioning of the wrist in slight extension facilitates anti-edema exercises and avoiding median nerve compression in the carpal tunnel.

**• Reduction of displaced DRFs**

**Figure 4:**
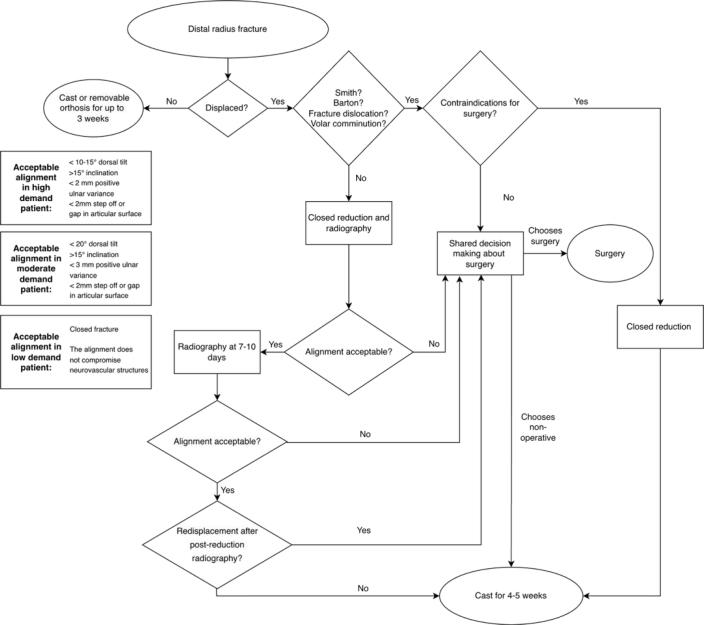
Proposed consensus treatment algorithm for DRF in the elderly.

Displaced fractures are reduced using manual traction and fracture mobilization to restore joint-surface alignment and ensure contact between the volar cortices. Care should be taken in the elderly to protect the skin during fracture reduction, both in the emergency department and in the operating room. A plaster cast is then applied, leaving the digits free. The reduction is confirmed by radiography. If in acceptable position—within limits for the patient’s functional demands—a follow-up radiographic examination is planned at 7–10 days. If an acceptable position cannot be achieved, treatment options should be discussed with the patient and a treatment algorithm as suggested is helpful (Figure 4).

**• Timing of surgery**

Timing is one key to successful surgical management. The longer the interval between injury and surgery, the greater the risk of inadequate fracture position at healing [58].

**• Techniques of internal fixation**

A VLP should be the primary implant of choice in open reduction and internal fixation. For very distal fractures, special distal plates or fragment-specific fixation are recommended. In cases of very comminuted fractures or severe osteoporotic bone, spanning plates could be advantageous. A preoperative CT scan is obtained if a volar rim or die-punch fracture is suspected. In high-energy fractures and fracture dislocations, assess for potential ligamentous instability. Reduce and pin the fracture to an anatomic position, then meticulously adjust the plate before securing it with screws.

If acceptable alignment cannot be maintained in patients with moderate or high functional demands, surgical treatment should be considered through a shared decision-making process [18]. Inherently unstable fractures such as Smith’s fractures (a fracture of the distal radius with volar displacement and/or tilt of the distal fragment) and Barton fractures (an intra‑articular fracture of the distal radius involving a rim of the articular surface, with associated dislocation or subluxation of the radiocarpal joint) are most often managed surgically. Additionally, dorsally displaced DRFs with volar, radial, or dorsal comminution carry a high risk of malunion, which must be carefully considered in treatment decisions for moderate and high functional demand patients [29].

### Outcome measures

In studies, PROMs are often used as the primary outcome, applying the concept of MCID to evaluate whether a treatment is of any clinical importance for the patient. PROMs such as the Quick Disabilities of the Arm, Shoulder, and Hand (QuickDASH) were developed as descriptive tools to evaluate chronic wrist and upper limb disorders and to assess longitudinal changes in response to treatment, rather than between-group comparisons [34]. The distribution is skewed in this population, and, because these are ordinal scales, using the mean and standard deviation to describe central tendencies and variation, and comparing mean group differences with the MCID may not be statistically appropriate. Even if the mean benefit is below the MCID limits, the proportion of patients with poor outcome may be obscured, which is higher in nonoperatively treated patients, as shown in a recent meta-analysis [6]. The MCID can be determined in several ways, with distribution-based and anchor-based methods being most used. Most studies report an MCID of 10–15 points for QuickDASH, though reported estimates have been found to range widely from 5 to 56 points, depending on the condition and population [35]. Regarding the Patient-Rated Wrist Evaluation (PRWE) the MCID has been estimated at 11.5 [36]. In both PROMs, the ceiling effect may limit the use of MCID for group comparisons as improvements measured by PROMs plateau within 1 year following DRF treatment [37], and small changes may go undetected with region specific PROMs such as the QuickDASH and PRWE [36].

### Advantages and disadvantages of non-surgical vs surgical management

In a recent systematic review and meta-analysis of all RCTs comparing VLP witho non-surgical management in adults, none favored cast treatment, while 8 of the 12 studies favored VLP for displaced DRFs [6]. The overall mean DASH score was 14 points for non-surgically treated patients and 5 points better for surgically treated patients (95% confidence interval [CI] 2.4–7.9). A 5-point difference in mean DASH is less than the commonly applied MCID limit of 10–15 scale steps. However, evidence remains limited due to a lack of clarity regarding what constitutes moderate and high functional demands, but it should be noted that chronological age is not always a reliable proxy for functional capacity.

### Non-surgical treatment in elderly individuals with DRFs

Non-displaced DRFs are treated with a dorsal cast. Displaced DRFs are reduced under local anesthesia using a hematoma block or under regional anesthesia, followed by a below-elbow plaster splint or circular cast [24]. If there is acceptable alignment, based on fracture measurements and patient characteristics and preferences, follow-up radiographs are obtained within 7–10 days to monitor re-displacement [18,19,22]. If alignment is maintained, casting is continued until 4–5 weeks after injury with clinical follow-up [16-22]. To reduce stiffness, the cast dressing or cast should not include the 1st carpometacarpal (CMC1) or the 4 metacarpophalangeal (MCP 2–4) joints. Furthermore, the wrist should be in a functional position, avoiding volar flexion or ulnar deviation [37]. The digits should be free, allowing a full range of motion (ROM), and the patient should be encouraged to perform anti-edema finger-pumping exercises. After cast removal, patients are encouraged to begin full active ROM of the wrist and weightbearing exercises over the following weeks.

**Take-home messages: Considerations in the choice of treatment**

- Discuss treatment options with the patient and assess their physical demands for hand function and the patient’s level of activity when advising treatment through a patient-centered decision approach.
- Individuals can be classified as high-, moderate-, and low-demand patients; alongside this, comorbidities, frailty, and cognitive function should be considered to assess their ability to tolerate surgery and adhere to a rehabilitation program.
- VLP is preferred in osteoporotic bone compared with pinning, external fixators, and fragment-specific fixation.
- Adverse events exist in both non-surgical and surgical treatment but differ in type. The patient should be informed about potential complications.

### Surgical treatment in elderly individuals with DRFs

Surgical treatment appears to enable earlier recovery of hand function and grip strength, as well as improved radiographic outcomes, but differences in functional outcomes tend to diminish by 1 year in older patients [6,38,39]. If there is an indication for surgery, it should be planned as a semi-acute operation. Surgical treatment of moderately displaced fractures is a good option for individuals with higher functional demands preferring a faster recovery, regardless of their chronological age [18]. To minimize complications, techniques should be adapted to optimize fixation in osteoporotic bone to avoid hardware failure [40]. Reduction maneuvers successful in younger bones may fail in cases with poor bone quality. Likewise, surgical techniques such as percutaneous pinning or external fixation, which may be effective for fractures in younger individuals with good bone quality, could be less effective in elderly individuals with DRFs [39]. External fixation compared with VLPs carries a higher risk of complications in very elderly individuals [41]. Several studies have shown that VLPs with angle-stable screws provide reliable subchondral support of the reduced joint surface, and good functional outcome also in elderly individuals [8,11,12,42-44].

### Surgical treatment in extra-articular and intra-articular fractures

For optimal stability, locking screws should be placed directly beneath the subchondral bone. Screws positioned more proximally in low-quality metaphyseal bone without subchondral support increase the risk of secondary displacement. Accurate plate positioning is particularly critical in osteoporotic bone, and distal screws must avoid penetrating the dorsal cortex to prevent extensor tendon injuries [45]. Even comminuted fractures of the radiocarpal joint surface can be safely stabilized in osteoporotic bone using standard VLPs [12,42].

### Considerations in severely comminuted fractures and fracture-dislocations

In severely comminuted fractures, volar plates may be insufficient. In non-osteoporotic bone, superior outcomes have been reported with fragment-specific fixation techniques that stabilize individual fragments until healing, when compared with external fixation [46]. However, multiple incisions and plates may increase the risk of complications with fragment-specific plates compared with treatment using a VLP [47]. In osteoporotic bone, the risk of hardware failure is increased, and the VLP is preferable to the external fixation due to fewer complications [41]. Still, external fixation is a treatment alternative in some comminuted DRF cases [48]. The wrist-spanning plate maintains proper hand-to-forearm alignment and may be a viable option [49]. Pronation and supination exercises can be initiated immediately, and finger mobility is facilitated by pain relief from fracture stabilization. The plate is usually removed after 3 months, allowing acceptable restoration of wrist flexion and extension.

### Distal radius fractures and concomitant ulnar metaphyseal fractures

DRFs combined with metaphyseal ulnar fractures represent a distinct injury pattern associated with poorer outcomes, and an increasing incidence is found with advancing age and osteoporosis [50,51]. While these injuries occur in approximately 6% of DRF patients overall [51], prevalence rises to nearly 20% in the population over 80 years [5]. PROMs are consistently worse with distal radius and ulnar metaphyseal fractures compared with isolated fractures of the distal radius [51,52], regardless of whether the distal ulna is treated non-surgically or surgically [51-54]. Key factors in managing these complex injuries include meticulous preoperative planning, a high index of suspicion for treatment failure, and a carefully considered informed decision as to whether to stabilize the ulnar fracture [55]. With the current lack of high-quality evidence regarding which fractures should be treated non-surgically or surgically, this decision is ideally made together with an experienced surgeon, as no general recommendation can yet be given.

### Complications following non-surgical and surgical treatment

A systematic review of DRFs in the elderly found no significant difference in complication rates between non-surgical management and VLP fixation [38]. Many studies comparing VLP fixation with cast immobilization in elderly patients with DRFs have reported high complication rates (Table) [8-12,15,42-45,59,60].

**Table:**
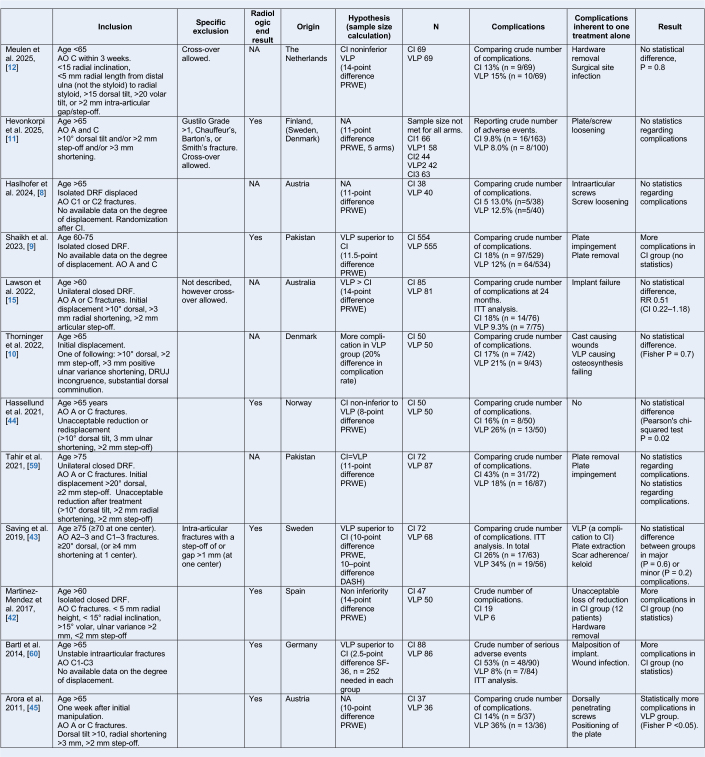
Complications and adverse events in the RCTs comparing cast immobilization (CI) with volar locking plates (VLP) in elderly patients

Non-surgical and surgical treatments share common complications, including malreduction, malunion, complex regional pain syndrome (CRPS), carpal tunnel syndrome (CTS), tenosynovitis, and tendon rupture [61,62]. Given that some complications are specific to the chosen treatment modality—such as infection or implant failure in surgical cases or subsequent corrective procedures in non-surgical cases—the clinical relevance of directly comparing these outcomes is difficult. Therefore, risk ratios should be interpreted with caution, as they may not fully capture the nuanced nature of treatment-specific risks.

A detailed overview of 12 RCTs on non-surgical vs surgical treatment is given in the Table.

### National treatment guidelines

To promote consistent, high-quality care, regardless of the treating physician or hospital, several national orthopedic associations have issued overarching recommendations on the preferred treatment.

The United Kingdom national guidelines from 2018 advise non-surgical treatment as the primary option for displaced DRFs in individuals over 65, unless there is significant bayonet deformity or neurological compromise. The guidelines also consider other factors, such as activity level, medical comorbidities, and fracture characteristics [16]. The 2020 North American guidelines recommend non-surgical treatment for individuals aged 65 years and older. Still, a patient-centered discussion to understand the patient’s functional demands is recommended in the decision-making process [17]. The Dutch guidelines from 2021 do not define age as a cut-off but recommend considering nonoperative treatment for displaced or re-displaced DRFs in individuals with dementia or advanced age [22]. In the Nordic countries, recommendations vary. A Danish treatment recommendation from 2023 suggested primarily non-surgical treatment of patients > 65 years with dorsally displaced DRFs [21], but emphasized that treatment should be individualized, considering functional level, comorbidities, and fracture morphology. The Norwegian national guidelines, published in 2015, recommend surgical treatment in individuals ≥ 18 years with unstable DRFs, except for those with a low functional level, defined by permanent inability to deal independently with day-to-day activities [20]. The Swedish national treatment guidelines from 2021 are based on radiographic parameters in combination with the patient’s functional needs [18]. Surgical treatment is indicated for a patient with high functional demands (“the need to use the wrist and hand in heavy labor or activities in work, free time or daily activities”), if 1 or more of the following radiological parameters after reduction are present: > 10° dorsal tilt, > 15° volar tilt, < 15° radial inclination, > 2 mm positive ulnar variance, > 2 mm intra-articular step-off, no volar cortical alignment, ulnar shift of the radius shaft, or incongruent distal radioulnar joint (DRUJ). In individuals with moderate functional demands (“the need to perform activities of daily living (ADL) independently, but without the need to load the wrist heavily in physical labor or leisure-time activities”), a greater displacement of the fracture is accepted, i.e., > 20° dorsal tilt, < 10° radial inclination, > 3 mm positive ulnar variance; the other radiological parameters are the same as for high-demand individuals. In those with low functional demands (“permanent incapability to perform ADLs independently”), surgical treatment is not recommended unless there is a skin lesion or concomitant neurovascular injury. Finland’s national guidelines, updated in 2023, recommend non-surgical treatment in displaced fractures in individuals > 65–70 years but emphasize shared decision-making in active elderly individuals [19].

### Osteoporosis screening and referral for treatment

DRFs are one of the commonest types of fragility fractures [63] and only a minority have normal bone mineral density (BMD) when assessed with dual-energy X-ray absorptiometry (DEXA). Because DRFs typically occur at a younger age than hip and vertebral fractures, they provide a unique opportunity to initiate osteoporosis screening and treatment, potentially reducing the subsequent risk of these fractures. Treatment of underlying abnormalities in bone metabolism may involve exercise and falls prevention strategies, calcium and vitamin D supplementation when indicated, bisphosphonates, calcitonin, receptor activator of nuclear factor kappa-B ligand (RANK-L) monoclonal antibodies (denusomab), recombinant parathyroid hormone (PTH), and parathyroid hormone-related protein (PTHrP) [64].

Initiating osteoporosis screening and treatment is crucial to reduce the risk of future fractures. Surgeons and physicians treating patients with DRFs are in the unique position to identify individuals at risk and to refer the patient to a Fracture Fragility Unit (FFU) or a Fracture Liaison Service (FLS).

### Health economic perspectives

Health economic evaluations can be conducted from either a healthcare provider or a societal perspective. From the provider’s perspective, direct medical expenses are included, whereas the societal perspective also incorporates costs such as productivity loss and the use of social services. To assess the value of a medical intervention, outcomes are expressed as QALYs (Quality-Adjusted Life Years), which combine the quantity and quality of life gained. Current evidence suggests that, at the group level, surgical treatment of the distal radius in the elderly is not cost-effective within 1 year of treatment compared with non-surgical [65,66]. In contrast, in the United States, a decision-tree analysis suggested that VLP surgery may be cost-effective in individuals aged 65 years and older [67]. There is a clear need for high-quality cost-effectiveness studies focused on this age group. Future research should carefully consider which costs to include, how best to measure health outcomes, and the appropriate time horizon to capture the actual value of each treatment, whether non-surgical or surgical.

### Choosing the treatment with an elderly patient

The goal of treating DRFs in elderly individuals should be to achieve outcomes that align with each patient´s functional needs and life goals, recognizing that priorities may range from maintaining independence to optimizing performance in specific activities. Ultimately, the treatment decision involves choosing between immediate surgical intervention and non-surgical management. The ultimate treatment decision should be in collaboration with the patient to align with their expectations [68,69]. Shared decision-making involves clearly explaining the potential benefits and risks of surgery compared with non-surgical treatment. Regardless of treatment choice, patients should be counseled regarding expected swelling, pain, and reduced range of motion following the fracture. These symptoms typically improve within the first 6 months but may continue to recover for several years [68]. Most elderly individuals tolerate malunion [33], and functional outcomes are comparable to those of the uninjured side; the long-term risk of osteoarthritis remains low, even 10 years after fracture, irrespective of treatment modality [70].

Individuals aged 70 and older are unlikely to achieve substantial benefit from surgery as a group [6]. Those with functional demands comparable to those of younger individuals may still benefit. An important priority, therefore, is to develop improved triage tools to identify individuals likely to benefit from surgical intervention vs those who can achieve satisfactory function despite moderate or even severe malunion.

**Figure 5:**
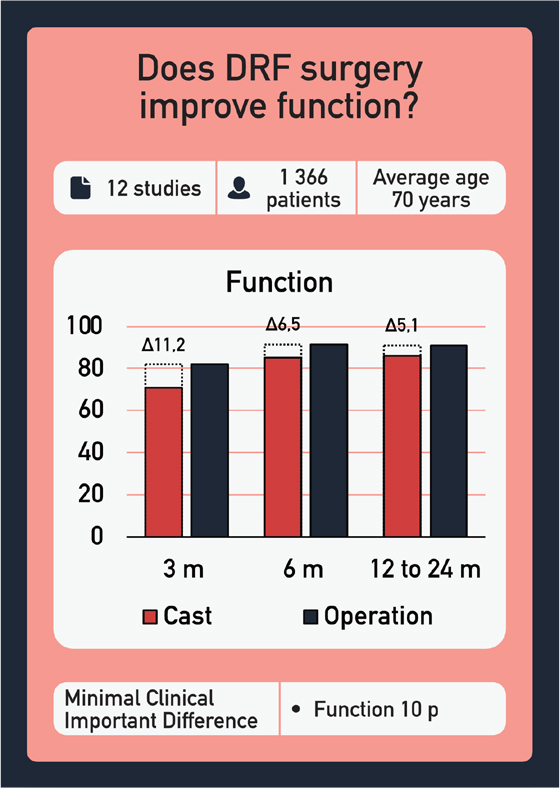
Does DRF surgery improve function? The inverse QuickDASH score, hence the higher the score (0 to 100) the better the outcome, as a representation for disability at 3, 6, 12, and 24 months after non-surgical treatment vs surgical treatment. The minimal clinically important difference (MCID) is set to 10 scale points.

**Figure 6:**
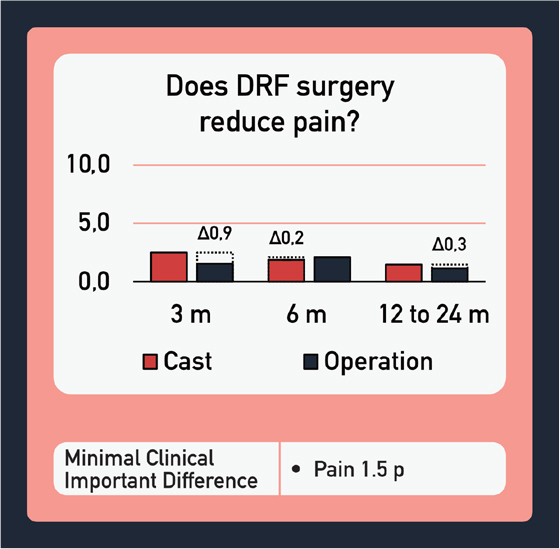
Does DRF surgery reduce pain? The Visual Analogue Scale (VAS) and Numeric Rating Scale (NRS) ranging from 0 to 10 at 3, 6, 12, and 24 months after non-surgical treatment vs surgical treatment; a lower score represents less pain. The MCID is set to 1.5 scale points.

**Figure 7:**
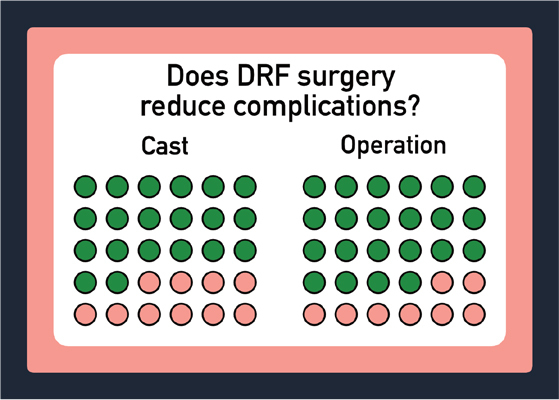
Does DRF surgery reduce the number of complications? A pink dot represents a patient with a complication, and a green dot indicates a patient without a complication in non-surgical treatment vs surgical treatment.

A treatment algorithm, as proposed in this article, may assist the treating physician in guiding these decisions (see Figure 4). Further, in a shared decision-making strategy, the physician could be aided by the decision-making graphics from a recent systematic review and meta-analysis (Figures 5 to 7). Functional definitions are necessary for patients most likely to benefit from surgical treatment. Instruments such as the Clinical Frailty Scale (CFS) have recently gained popularity [71] and could assist clinicians in classifying older individuals by their functional status in determining who may benefit from surgical treatment. The CFS is preferable to chronological age as a triage and decision-aid tool [72], but, to date, no studies have evaluated its applicability for DRFs.

## Note

Figures 1–3. Frequent radiographic measurements used in the assessment of a distal radius fracture. In this example a minimally displaced, extra-articular distal radius fracture after reduction and dorsal cast in a 76-year-old female patient with a moderate level of function.

## Notes

Handling co-editor: Cecilia Rogmark

*Acta* thanks Sina Hulkkonen and Yngvar Krukhaug for help with peer review of this manuscript.
